# Associations between self‐stigma and socio‐clinical characteristics among patients with bipolar disorder in Japan

**DOI:** 10.1002/pcn5.70366

**Published:** 2026-06-18

**Authors:** Mayuko Ishigaki, Dai Kezuka, Yuko Isogaya, Eiji Suzuki

**Affiliations:** ^1^ Department of Psychiatry Tohoku Medical and Pharmaceutical University Hospital Sendai Japan; ^2^ Division of Psychiatry Tohoku Medical and Pharmaceutical University Sendai Japan

Self‐stigma, also referred to as internalized stigma, is described as an individual's acceptance and incorporation of negative social stigmas and stereotypes pertaining to mental illness.^1^ Particularly, self‐stigma among patients with bipolar disorder (BD) is reportedly more severe than in patients with major depressive or anxiety disorders.^2^, ^3^ Although global research on BD notes a relationship between self‐stigma and various socio‐clinical factors,^4^, ^5^ research on self‐stigma in Asian patients with BD remains limited. Therefore, this study investigates the association between self‐stigma and socio‐clinical characteristics among patients with BD in Japan.

To this end, we conducted a questionnaire survey from January to March 2024 among 528 members of the Japanese Alliance of Bipolar Disorder—a self‐help group for BD in Japan.^6^, ^7^ Of these, 163 were included in the final statistical analyses. The questionnaire captured social aspects, including age, sex, employment status, and marital status, as well as clinical aspects, such as duration since BD diagnosis, history of psychiatric hospitalization, and history of suicide attempts. Moreover, self‐stigma was assessed using the Japanese version of the 10‐item Internalized Stigma of Mental Illness (ISMI).^8^ The scale comprised 10 items, each to be rated from “1 = Strongly disagree” to “5 = Strongly agree,” with higher total scores indicating more severe self‐stigma (Cronbach's *α* = 0.84). For identification of the socio‐clinical factors associated with self‐stigma, a multiple regression analysis was performed using the total ISMI score as the dependent variable, with social and clinical factors included as the independent variables. Statistical analyses were performed using IBM SPSS Statistics version 21.0.

The participants comprised 83 females and 80 males, with a mean age of 50.69 years. As shown in Table 1, the multiple regression analysis revealed that being male (*β* = 0.18, *p* < 0.05), unemployed (*β* = 0.28, *p* < 0.01), and possessing a history of suicide attempts (*β* = 0.17, *p* < 0.05) were significantly associated with higher ISMI scores. On the contrary, a longer duration since BD diagnosis (*β* = −0.21, *p* < 0.05) was significantly associated with lower ISMI scores.

**Table 1:** Multiple regression analysis of socio‐clinical characteristics associated with Japanese version of the Internalized Stigma of Mental Illness scale (ISMI) scores among patients with bipolar disorder in Japan (*N* = 163).

	*B*	SE	*β*	95% CI	*p* value	VIF
Age	−0.49	0.06	−0.08	−0.17 to 0.07	0.42	1.67
Sex (male)	2.73	1.13	0.18	0.51 to 4.96	**<0.05**	1.03
Employment status (unemployed)	4.52	1.28	0.28	2.01 to 7.05	**<0.01**	1.14
Marital status (single/divorced)	0.10	1.17	0.06	−1.18 to 3.25	0.36	1.08
Duration since BD diagnosis	−0.016	0.01	−0.21	−0.029 to −0.002	**<0.05**	1.59
History of psychiatric hospitalization	1.44	1.25	0.09	−1.01 to 3.89	0.25	1.23
History of suicide attempts	2.63	1.24	0.17	0.21 to 5.08	**<0.05**	1.24

*Note*: Statistically significant values are in bold. The multiple regression model showed statistical significance: adjusted *R* ^2^ = 0.13, *F* (7, 155) = 4.33, *p* < 0.01.

Abbreviations: *β*, standardized coefficients; *B*, unstandardized coefficients; BD, bipolar disorder; CI, confidence interval; SE, standard error; VIF, variance inflation factor.

Overall, consistent with previous studies,^4^, ^5^ this study suggests that self‐stigma is elevated among patients who are unemployed, possess a history of suicide attempts, and are in earlier stages of BD diagnosis. These findings highlight the importance of employment support and treatment for suicidal ideation, not only for direct clinical benefits but also for reducing self‐stigma. In particular, structured anti‐stigma interventions should be integrated into standard early‐stage treatment for patients with BD in Japan. This study also emphasizes the need for intensive interventions—such as psychoeducation on self‐stigma—at the time of or shortly after the diagnosis of BD.

Furthermore, this study indicates that self‐stigma tends to be greater in males than in females. Systematic reviews have reported inconsistent findings regarding sex‐related differences in self‐stigma.^1^ Nevertheless, men reportedly possess a stronger social stigma pertaining to mental illness owing to greater exposure to and familiarity with mental illness among women.^9^ Particularly in Japan, social stigma remains widespread, accelerated by the severity of mental illness.^10^ One possible explanation for this is that Japanese men possessing higher levels of social stigma—which functions as the foundation for self‐stigma—are likely to develop even stronger self‐stigma when diagnosed with BD. In addition, traditional gender roles in Japan, particularly expectations for men as breadwinners, may partly explain the observed sex differences, given the association between unemployment and higher self‐stigma.

To the best of our knowledge, this study is the first in Japan to assess self‐stigma among patients with BD. Accordingly, the clinical significance thereof arises in that a new and essential psychological perspective—self‐stigma—is introduced and related to the treatment of BD; specifically, patients with BD are identified as requiring more comprehensive support and care for reducing self‐stigma based on socio‐clinical characteristics.

Notwithstanding, this study possesses several limitations. First, the cross‐sectional survey design rendered the inference of causal relationships among the variables difficult. Second, the adjusted *R* ^2^ value of 0.13 suggests that unmeasured factors may contribute to self‐stigma. Because current mood states were not assessed, temporary symptom‐related effects could not be distinguished from more stable trait‐like self‐stigma. Therefore, unemployment may partly reflect current syndromal severity rather than solely a social determinant. Third, there exists potential for selection bias. Members of self‐help groups may differ from the general population of patients with BD, as they may have higher levels of mental health literacy or, conversely, may participate due to experiencing particularly high levels of stigma or distress. Therefore, caution is required when generalizing the present findings to all patients with BD.

## AUTHOR CONTRIBUTIONS

Eiji Suzuki and Mayuko Ishigaki designed the study, collected, analyzed, and interpreted the data, and prepared and edited the manuscript. Dai Kezuka and Yuko Isogaya analyzed and interpreted the data and prepared and edited the manuscript. All the authors have read and approved the final version of the manuscript.

## CONFLICT OF INTEREST STATEMENT

Eiji Suzuki received scholarship (encouragement) donations from Eisai Co., Ltd., Shionogi & Co., Ltd., Daiichi Sankyo Co., Ltd., Takeda Pharmaceutical Company Limited, and Mochida Pharmaceutical Co., Ltd., and Lecture fees from Otsuka Pharmaceutical Co., Ltd., MSD K.K., Eisai Co., Ltd., and Lundbeck K.K. The other authors have no conflicts of interest to declare.

## ETHICS APPROVAL STATEMENT

This study was approved by the Clinical Research Review Committee of Tohoku Medical and Pharmaceutical University Hospital (research registration no. 2024‐2‐001). The study was explained in writing at the time of the mail survey, and written informed consent was obtained from the participants. Participants could withdraw their consent at any time without prejudice.

## PATIENT CONSENT STATEMENT

Informed consent has been obtained from all individuals included in this study.

## CLINICAL TRIAL REGISTRATION

N/A.

## Data Availability

The data supporting the findings of this study are available from the corresponding author, Dai Kezuka, upon reasonable request.
